# miR-19a-3p enhances TGF-β1-induced cardiac fibroblast activation *via* targeting BAMBI

**DOI:** 10.7555/JBR.37.20230313

**Published:** 2024-05-29

**Authors:** Pengxi Shi, Ao Tan, Yuanyuan Ma, Lingli Que, Chuanfu Li, Yongfeng Shao, Haoliang Sun, Yuehua Li, Jiantao Li

**Affiliations:** 1 Key Laboratory of Targeted Intervention of Cardiovascular Disease, Collaborative Innovation Center for Cardiovascular Disease Translational Medicine, School of Basic Medical Science, Nanjing Medical University, Nanjing, Jiangsu 211166, China; 2 Department of Surgery, East Tennessee State University, Johnson City, TN 37614-0575, USA; 3 Department of Cardiovascular Surgery, the First Affiliated Hospital of Nanjing Medical University, Nanjing, Jiangsu 210029, China

**Keywords:** miR-19a-3p, BAMBI, TGF-β1, SMAD2/3, myocardial fibrosis

## Abstract

Myocardial fibrosis is a major pathogenic factor contributing to cardiac remodeling and heart failure. Recent research has indicated that microRNAs play a crucial role in the progression of cardiac fibrosis. Bone morphogenetic protein and activin membrane-bound inhibitor (BAMBI) have been shown to alleviate myocardial fibrosis by inhibiting the transforming growth factor β1 (TGF-β1) signaling pathway. Therefore, the current study aimed to elucidate the post-transcriptional regulation of BAMBI by miR-19a-3p and its role in TGF-β1-induced cardiac fibroblast activation. We found that transverse aortic constriction induced both myocardial interstitial and perivascular collagen deposition. Quantitative reverse transcription-PCR (qRT-PCR) analysis showed that the expression level of miR-19a-3p was increased in the myocardial tissues of cardiac fibrosis, and TGF-β1 induced an upregulation in miR-19a-3p expression in cardiac fibroblasts. The dual-luciferase reporter assay and qRT-PCR verified that miR-19a-3p directly bound to the 3′ untranslated regions of *BAMBI* mRNA, thereby reducing BAMBI expression and diminishing its ability to inhibit the TGF-β1 signaling pathway. Furthermore, overexpression of miR-19a-3p mimic increased the activation of TGF-β1/SMAD2/3 pathway signaling, promoting cardiac fibroblast activation. However, this activation was blocked by BAMBI overexpression. These findings imply that miR-19a-3p enhances the activation of TGF-β1/SMAD2/3 by inhibiting BAMBI, further boosting the activation of cardiac fibroblasts and contributing to myocardial fibrosis.

## Introduction

Myocardial fibrosis is a major contributor to aberrant cardiac remodeling and plays a key role in pathological alterations associated with chronic heart disease induced by pressure overload^[1]^. Heart failure develops because of excessive extracellular matrix (ECM) accumulation, which reduces ventricular compliance^[2]^. The principal effector cells causing fibrosis are cardiac fibroblasts (CFs)^[3]^. When stimulated, CFs transdifferentiate into myofibroblasts and secrete ECM between myocytes^[4]^. This process involves various cytokines, chemokines, and growth factors, resulting in an excessive ECM and an imbalance in the collagen ratio, which eventually leads to cardiac fibrosis. Unfortunately, no effective treatment currently exists to halt the progression of cardiac fibrosis.

Over the past few decades, mounting data have established that the TGF-β1/SMADs signaling pathway is a key driver of fibrosis and is activated when cardiac pressure is overloaded^[5]^. Specifically, active TGF-β1 binds to its receptor, which phosphorylates the C-terminal serine residue of receptor-regulated Smads (R-Smads, including SMAD2 and SMAD3). Phosphorylated SMAD2/3 then forms a transcription complex with SMAD4, which transfers into the nucleus to drive target gene transcription. TGF-β1 is a primary cytokine that leads to CF activation, triggering the TGF-β1 signaling in fibroblasts through SMAD3, promoting α-smooth muscle actin (α-SMA) transcription and collagen production^[6]^. Thus, it is possible to decrease proliferation and differentiation in CFs by inhibiting the TGF-β1 signaling pathway, thereby attenuating the progression of cardiac fibrosis.

The bone morphogenetic protein and activin membrane-bound inhibitor (BAMBI) is a transmembrane glycoprotein composed of 260 amino acids, including an intracellular C-terminus, a transmembrane segment, and an extracellular ligand-binding domain at the N-terminus. BAMBI resembles the type Ⅰ receptors of the TGF-β family in the extracellular domain^[7]^. Intriguingly, the intracellular region of BAMBI lacks the serine/threonine kinase domain, rendering it unable to phosphorylate the R-SMADs in the cytoplasm. Consequently, this leads to an attenuated response to TGF-β signaling by acting as a dominant-negative pseudo-receptor that disrupts the activation of downstream signaling pathways. This unique mechanism underscores its role as a critical regulator of TGF-β signaling. Importantly, studies have demonstrated that aberrant expression of BAMBI plays a significant role in the pathophysiology of inflammation, fibrosis, and cancer progression. For instance, inhibiting BAMBI expression promotes the TGF-β/SMADs signaling pathway and prevents colon cancer metastasis^[8]^. Thus, BAMBI may alleviate cardiac fibrosis by inhibiting the TGF-β signaling pathway and reducing collagen formation.

MicroRNAs are small noncoding RNAs, with a length of 21–24 nucleotides, that regulate protein-coding gene expression by targeting messenger RNAs (mRNAs)^[9]^. Through imperfect complementary base pairing, these molecules bind to the 3′ untranslated regions (UTR) of target mRNAs, leading to mRNA degradation and gene silencing^[10]^. This post-transcriptional regulation is directly related to the activation of numerous signaling pathways and the progression of various diseases. For instance, excessive ventricular hypertrophy and heart failure have been associated with miR-133 overexpression^[11]^. miR-19a, a key member of the miR-17-92 cluster (miR-17/18a/19a/19b/20a/92a), plays a significant role in disease progression, including cancer and fibrosis^[12]^. Specifically, miR-19 promotes lung cancer by decreasing the expression levels of E-cadherin and inducing EMT in H1299 cells^[13]^. Furthermore, miRNA-target analysis revealed that the binding site for miR-19a-3p at the 3′ UTR of *BAMBI* mRNA is highly conserved across species, indicating a crucial role in the development of fibrosis.

In the current study, we aimed to identify the role of miR-19a-3p in the TGF-β1-induced cardiac fibroblast activation, which may provide a novel therapeutic target for myocardial fibrosis.

## Materials and methods

### Human tissue sampling

Human myocardium samples were obtained from organ donors or patients with hypertrophic cardiomyopathy undergoing cardiac valve replacement surgery. A written informed consent was obtained from each patient or family member of the donor. The study was approved by the Institutional Review Board of the First Affiliated Hospital of Nanjing Medical University (Approval No. 2019-SR-300) and conducted in accordance with the ethical guidelines of the 1975 Declaration of Helsinki. Detailed sample information is shown in ***Table 1***.

**Table 1:** General information of human samples

Groups	Age	Sex	Etiology
Donation	57	Male	Accidental death
Donation	47	Female	Accidental death
Donation	50	Male	Accidental death
Donation	17	Male	Accidental death
Donation	48	Female	Accidental death
HCM	57	Female	HCM
HCM	47	Male	HCM
HCM	58	Female	HCM
HCM	43	Male	HCM
HCM	28	Male	HCM
Abbreviation: HCM, hypertrophic cardiomyopathy.			

### Animals

Both Sprague-Dawley rats (1–3 days old), regardless of sex, and male C57BL/6J mice (eight weeks old) were obtained from the Animal Core Facility of Nanjing Medical University (Nanjing, China). All animal research was approved by the Animal Care and Use Committee of Nanjing Medical University (Approval No. IACUC 1811030), and was conducted following the National Research Council's Guide for the Care and Use of Laboratory Animals.

### Construction of transverse aortic constriction (TAC) in mice

Male C57BL/6J mice (8 weeks, 22–25 g) were anesthetized with 1.5% isoflurane inhalation. The TAC operation was carried out exactly as previously described^[14]^. Briefly, the aortic arch was exposed, and a 27-gauge needle was placed alongside the transverse aorta. The needle was then ligated with the artery using a 5-0 silk suture and withdrawn immediately, leaving the aortic arch confined to the needle's diameter. A similar procedure was employed for the sham operation; however, the suture was not tied around the aorta.

### Masson's trichrome staining

The heart samples from each group were fixed in a 4% polyformaldehyde solution. They were embedded in paraffin (Cat. #A6330, Millipore, Billerica, MA, USA) for 24 h and sliced at 5-μm thickness. To detect cardiac collagen deposition, the tissues were counterstained using hematoxylin, ponceau, and toluidine blue (Cat. #G1340, Solarbio, Beijing, China), and observed using brightfield microscopy (BX51, OLYMPUS, Tokyo, Japan).

### Cell culture

#### Isolation and treatment of the primary newborn rat ventricular cardiac fibroblasts (NRCFs)

NRCFs were obtained from Sprague-Dawley rats that were one to three days old, as described previously^[15]^. NRCFs were cultured in Dulbecco's Modified Eagle Medium (DMEM, Cat. #12800-017, Life Technologies Corporation, Gaithersburg, MD, USA) supplemented with 10% fetal bovine serum (FBS, Cat. #04-001-1A, Biological Industries, Kibbutz Beit Haemek, Israel) after being plated at a density of 10^6^ cells per milliliter. NRCFs were passaged at a confluence of 80% to 90%. The second passage of NRCFs was used for formal experiments^[16–17]^.

We used miR-19a-3p mimic or inhibitor, along with their respective negative controls (NC) (RiboBio, Guangzhou, China) to investigate the function of miR-19a-3p in cardiac fibroblast activation. NRCFs were seeded in Opti-MEM, and transfected with 50 nmol/L mimic (or 50 nmol/L NC for mimic) or 100 nmol/L inhibitor (or 100 nmol/L NC for inhibitor) using Lipofectamine 2000 (Cat. #11668-019, Invitrogen, Carlsbad, CA, USA) at a 70% to 80% confluence. The transfection mixture was refreshed 6 h later with DMEM supplemented with 10% FBS. After 48 h of incubation, NRCFs were stimulated with TGF-β1 (10 ng/mL; Cat. #100-21C-10UG, PeproTech, Rocky Hill, NJ, USA) in DMEM containing 1% FBS for another 24 h.

#### Cell culture of HT1080 and HEK 293T cells

Both HEK 293T and HT1080 cell lines were obtained from the Cell Bank, Chinese Academy of Sciences, with their authenticity verified. Cells were cultured in DMEM supplemented with 10% FBS in a cell incubator with 5% CO_2_. All cell lines used in the study were tested and confirmed to be free of *Mycoplasma*.

At a confluence of 80%, HT1080 cells were placed in Opti-MEM and transfected with a mixture of 50 nmol/L miR-19a-3p mimic (or 50 nmol/L NC for mimic) and 2 μg pcDNA3.1-*BAMB*I-3×Flag (or 2 μg pcDNA3.1-GFP) (Miaoling Biology, Wuhan, China) using Lipofectamine 2000. Six hours after the transfection, the culture medium was refreshed with DMEM supplemented with 10% FBS. After 48 h of incubation, TGF-β1 (10 ng/mL) was used to treat HT1080 cells for another 24 h.

### Western blotting

Cells were harvested using RIPA buffer (Cat. #P0013B, Beyotime, Beijing, China), and the protein concentration was determined using a BCA protein assay kit (Cat. #23225, Thermo Fisher Scientific, Waltham, MA, USA). Proteins were denatured and then prepared for Western blotting analysis. Proteins were separated by SDS-PAGE and transferred to a PVDF membrane using a wet transfer system. The membrane was blocked using 5% non-fat dry milk in tris-buffered saline with Tween 20 (TBST) for 1 h, then incubated with antibody overnight at 4 ℃. After washing with TBST, the membrane was incubated with an HRP-conjugated secondary antibody for 1 h at room temperature. The signals were detected using the enhanced chemiluminescent system (Pierce, Rockford, IL, USA) and quantified by scanning densitometry with an Image Lab analysis system (Bio-Rad, Hercules, CA, USA).

Antibodies targeting the following proteins were used in the current study: p-SMAD2 (S465/467)/SMAD3 (E423/425) (1∶1000; Cat. #8828S, Cell Signaling Technology, Danvers, MA, USA), SMAD2/3 (1∶1000; Cat. #8685S, Cell Signaling Technology), BAMBI (1∶1000; Cat. #ab203070, Abcam, Cambridge, MA, USA), α-SMA (1∶1000; Cat. #A5228, Sigma-Aldrich, St. Louis, MO, USA), GAPDH (1∶1000; Cat. #AF0006, Beyotime), β-tubulin (1∶1000; Cat. #AF1216, Beyotime), collagen type Ⅰ alpha 2 polyclonal antibody (1∶1000; Cat. #14695-1-AP, Proteintech, Chicago, IL, USA).

### RNA extraction and quantitative reverse transcription-PCR (qRT-PCR)

The total RNA was extracted from cells using the RNA isolator Total RNA Extraction Reagent (Cat. #R401-01-AA, Vazyme, Nanjing, China). RNA was reverse-transcribed into cDNA using the PrimeScript RT Reagent Kit with gDNA Eraser (Cat. #RR047A, Takara, Tokyo, Japan) according to the manufacturer's protocol. MicroRNAs were reverse-transcribed using the miRNA 1st Strand cDNA Synthesis Kit (by stem-loop) (Cat. #MR101-02, Vazyme) following the manufacturer's instructions. Real-time PCR was performed on a StepOnePlus Real-Time PCR System (Thermo Fisher Scientific). The Bulge-Loop hsa-miR-19a-3p Primer Set (Cat. #MQPS0000773-1-200, RiboBio) was used to quantify miR-19a-3p levels. The primers for detecting mRNA expression levels were synthesized by Invitrogen. *Hprt* and *U6* served as internal controls for mRNA and microRNA, respectively. The relative expression level of RNA was calculated using the 2^−ΔΔCT^ method. The primer sequences are listed in ***Table 2***.

**Table 2:** List of primer sequences

Genes	Forward (5ʹ-3ʹ)	Reverse (5ʹ-3ʹ)
Rat *Hprt*	GTTGGATACAGGCCAGACTTTGTT	GATTCAACTTGCGCTCATCTTAGGC
Rat *Col1a2*	TGTCGATGGCTGCTCCAAAA	CCGATGTCCAGAGGTGCAAT
Rat *Col3a1*	TGGGAAAGGTGAAATGGGTCC	ATTCCTCCCACTCCAGACTTG
Mus *Hprt*	GTTGGATACAGGCCAGACTTTGTT	GATTCAACTTGCGCTCATCTTAGGC
Mus *Col1a2*	AATGGTGGCAGCCAGTTTGA	TCCAGGTACGCAATGCTGTT
Mus *Col3a1*	TGACTGTCCCACGTAAGCAC	GAGGGCCATAGCTGAACTGA
Mus *Tgfb1*	ACTGGAGTTGTACGGCAGTG	GGGGCTGATCCCGTTGATTT
Mus *Acta2*	CTTCGTGACTACTGCCGAGC	AGGTGGTTTCGTGGATGCC
Hsa *HPRT*	CCTGGCGTCGTGATTAGTGA	CGAGCAAGACGTTCAGTCCT
Hsa *COL1A2*	GCCTAGCAACATGCCAATC	GCAAAGTTCCCACCGAGA
Hsa *COL3A1*	TGAAGGGCAGGGAACAAC	GAGGGCGAGTAGGAGCAGT
Abbreviations: *Hprt*, hypoxanthine phosphoribosyltransferase; *Col1a2*, collagen type Ⅰ alpha 2 chain; *Col3a1*, collagen type Ⅲ alpha 1 chain; *Tgfb1*, transforming growth factor beta 1; *Acta2*, actin alpha 2, smooth muscle.		

### Dual-luciferase reporter assay

The 3′ UTR sequence of *BAMBI*, encompassing the predicted binding site for miR-19a-3p, was amplified and subsequently cloned downstream of the firefly luciferase gene in the pGL3 vector (Miaoling Biology, Wuhan, China). To establish a NC, a mutant of the *BAMBI* 3′ UTR was generated by introducing specific mutations within the miR-19a-3p seed region. HEK293T cells were co-transfected with 50 ng of either the wild-type (WT) or mutant *BAMBI* 3′ UTR reporter constructs, along with 50 nmol/L of either the miR-19a-3p mimic or a NC mimic using Lipofectamine 2000. Additionally, 10 ng of the pTK-Rluc vector (Miaoling Biology), encoding Renilla luciferase, was co-transfected as an internal control to normalize for variations in transfection efficiency. Following an incubation period of 24 h, cells were washed twice with phosphate-buffered saline. According to the manufacturer's protocol, the Dual-Luciferase Reporter Assay System (Cat. #E1910, Promega Corporation, Madison, WI, USA) was used to detect relative luciferase activity.

### Immunofluorescence staining

NRCFs or sections were fixed in 4% buffered paraformaldehyde at room temperature for 30 min, then permeabilized with 0.3% Triton X-100 at room temperature for another 30 min, and blocked using 10% BSA for 30 min. Subsequently, the cells or sections were incubated with primary antibodies at 4 ℃ overnight, followed by incubation with Alexa Fluor 594 donkey anti-mouse antibody (Cat. #R37115, Invitrogen, 1∶500) or Alexa Fluor 488 donkey anti-rabbit antibody (Cat. #R37116, Invitrogen, 1∶500) at 37 ℃ for 1 h. Fluorescence images were captured using a fluorescent microscope.

### Proliferation assay

The proliferation of NRCFs was assessed using the 5-ethynyl-2-deoxyuridine (EdU) kit (Cat. #C0078S, Beyotime) following the manufacturer's instructions. The proliferation rate was calculated by comparing the quantity of EdU-positive cells to the total number of Hoechst-stained cells.

### Wound scratch assay

NRCFs were seeded in a six-well plate and then transfected and treated. Cell monolayers were scratched using 200 μL plastic pipettes. An inverted light microscope was used to monitor the migration of NRCFs into the cell-free area 24 h after scratching. The migration rate was calculated as [1 − (residual scratch width/original scratch width)] × 100%.

### Statistical analysis

GraphPad Prism 9 (GraphPad Software, San Diego, CA, USA) was used for statistical analysis and figure generation. Data were presented as means ± standard deviation. Comparisons between two groups were performed using a two-tailed unpaired Student's *t*-test under the condition of homogeneity of variance (P > 0.1). Otherwise, an unpaired Student's *t*-test with Welch's correction was used. For comparisons among three or more groups, one-way ANOVA analysis was used. A *P*-value < 0.05 was considered statistically significant.

## Results

### The expression of miR-19a-3p was increased in cardiac fibrosis

**Figure 1:**
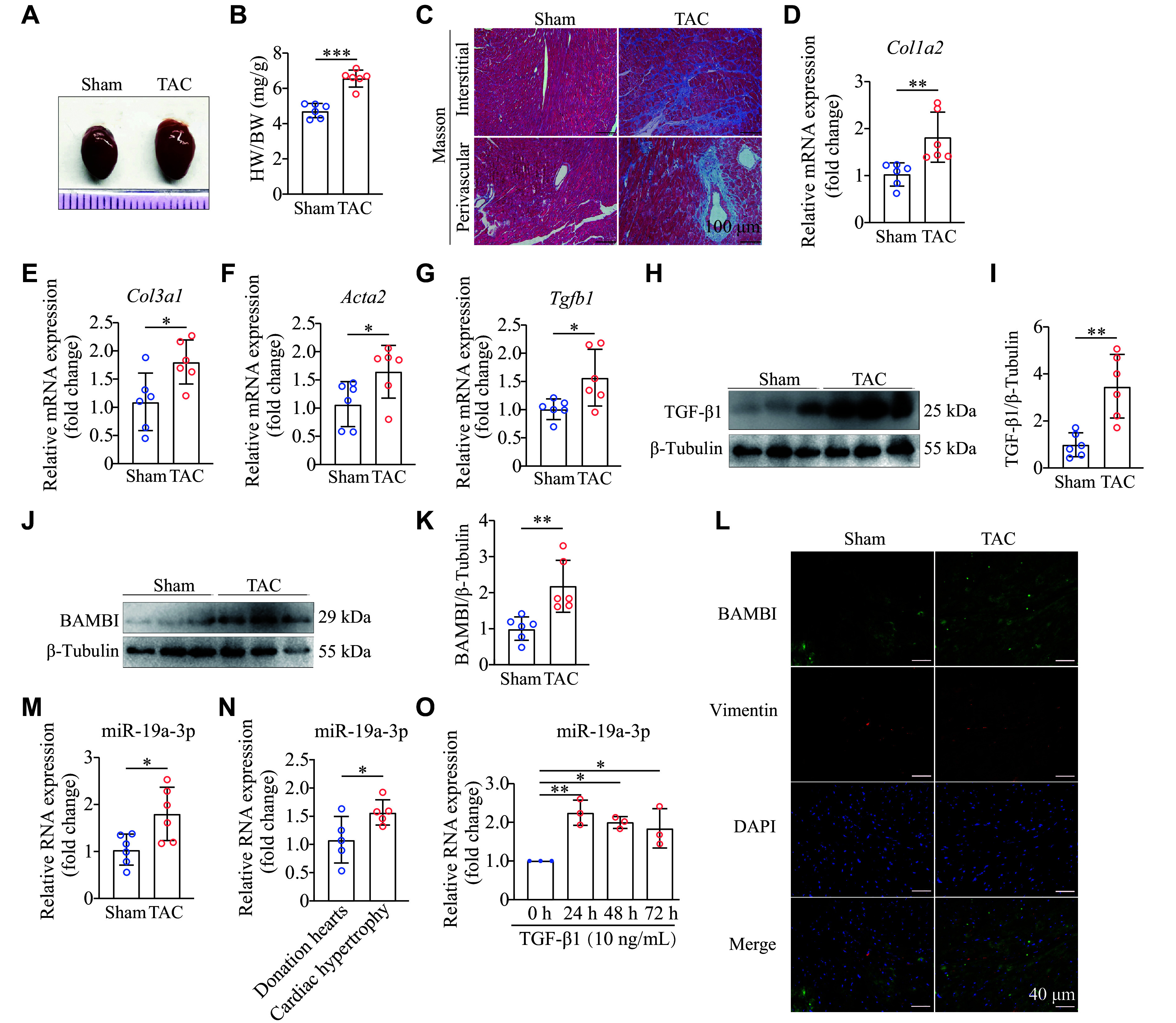
miR-19a-3p levels were increased in the myocardium of cardiac fibrosis and the TGF-β1-induced NRCFs. A–M: Eight-week-old male mice were subjected to transverse aortic constriction (TAC) or sham surgery for four weeks. A: Representative gross morphology of the hearts. Scale bar, 1 mm. *n* = 6. B: Heart weight-to-body weight ratio (HW/BW) of the mice. *n* = 6. C: Representative graphs of Masson's trichrome staining. Scale bar, 100 μm. *n* = 6. D–G: Quantitative reverse transcription-PCR (qRT-PCR) was performed to analyze the expression of fibrotic markers, including *Col1a2* (D), *Col3a1* (E), *Acta2* (F), and *Tgfb1* (G) using *Hprt* as the internal reference. *n* = 6. H–K: Protein levels of TGF-β1 (H and I) and BAMBI (J and K) in sham- or TAC-treated mouse hearts were detected by Western blotting. The grayscale values of protein bands were statistically analyzed and normalized to the internal control, β-tubulin. *n* = 6. L: Immunofluorescence staining of vimentin and BAMBI in sham- or TAC-treated mouse hearts. Scale bar, 40 μm. M: The levels of miR-19a-3p in sham- or TAC-treated mouse hearts were examined by qRT-PCR. *n* = 6. N: The levels of miR-19a-3p in heart tissues from five patient hearts with cardiac hypertrophy and five normal hearts from donations were detected by qRT-PCR. *n* = 5. O: NRCFs were cultured with TGF-β1 (10 ng/mL) for 24, 48, and 72 h. The levels of miR-19a-3p were then examined by qRT-PCR. *n* = 3. *U6* served as the internal reference (M–O). Data are presented as mean ± standard deviation. Statistical analyses were performed by Student's *t*-test. ^*^P < 0.05 and ^**^P < 0.01. Abbreviations: NRCFs, newborn rat ventricular cardiac fibroblasts; TGF-β1, transforming growth factor β1; BAMBI, bone morphogenetic protein and activin membrane-bound inhibitor; GAPDH, glyceraldehyde 3-phosphate dehydrogenase; DAPI, diamidino-phenyl-indole.

Cardiac pressure overload may result in left ventricular fibrosis and, ultimately, heart failure. We subjected the mice to TAC for four weeks and examined the profibrotic signal levels in the murine left ventricle. Notably, TAC-induced cardiac hypertrophy was observed in WT mice (***Fig. 1A***). A significant increase in the heart weight-to-body weight ratio was also observed in the TAC group, compared with the sham group (***Fig. 1B***). Masson's trichrome staining showed that TAC caused both myocardial interstitial and perivascular collagen deposition (***Fig. 1C***). Consistently, mRNA expression levels of collagen and fibroblast activation markers (*Col1a2*, *Col3a1*, *Acta2*, and *Tgfb1*; ***Fig. 1D***–***1G***) were significantly elevated in the left ventricle of TAC mice, compared with those in the sham group. After four weeks of TAC surgery, protein levels of ΤGF-β1 and BAMBI were significantly increased (***Fig. 1H***–***1K***). Additionally, fluorescence co-localization analysis showed that BAMBI levels were significantly upregulated in mouse fibroblasts after TAC (***Fig. 1L***). Surprisingly, miR-19a-3p expression levels were significantly increased in pressure overload-induced fibrosis in mice (***Fig. 1M***). Moreover, qRT-PCR analysis of human heart tissues showed that expression levels of miR-19a-3p were also significantly upregulated in hearts with cardiac hypertrophy (***Fig. 1N***). Collectively, these results suggest that miR-19a-3p may play a role in cardiac fibrosis.

TGF-β1 is a known contributor to myocardial fibrosis. To investigate the involvement of miR-19a-3p in cardiac fibroblast activation, NRCFs were exposed to 10 ng/mL of TGF-β1 for various durations. qRT-PCR analysis revealed that miR-19a-3p levels were significantly higher in TGF-β1-treated NRCFs than in control cells (***Fig. 1O***). Collectively, these results suggest that TGF-β1 may induce the expression of miR-19a-3p in NRCFs.

### miR-19a-3p promoted TGF-β1-induced NRCF differentiation

**Figure 2:**
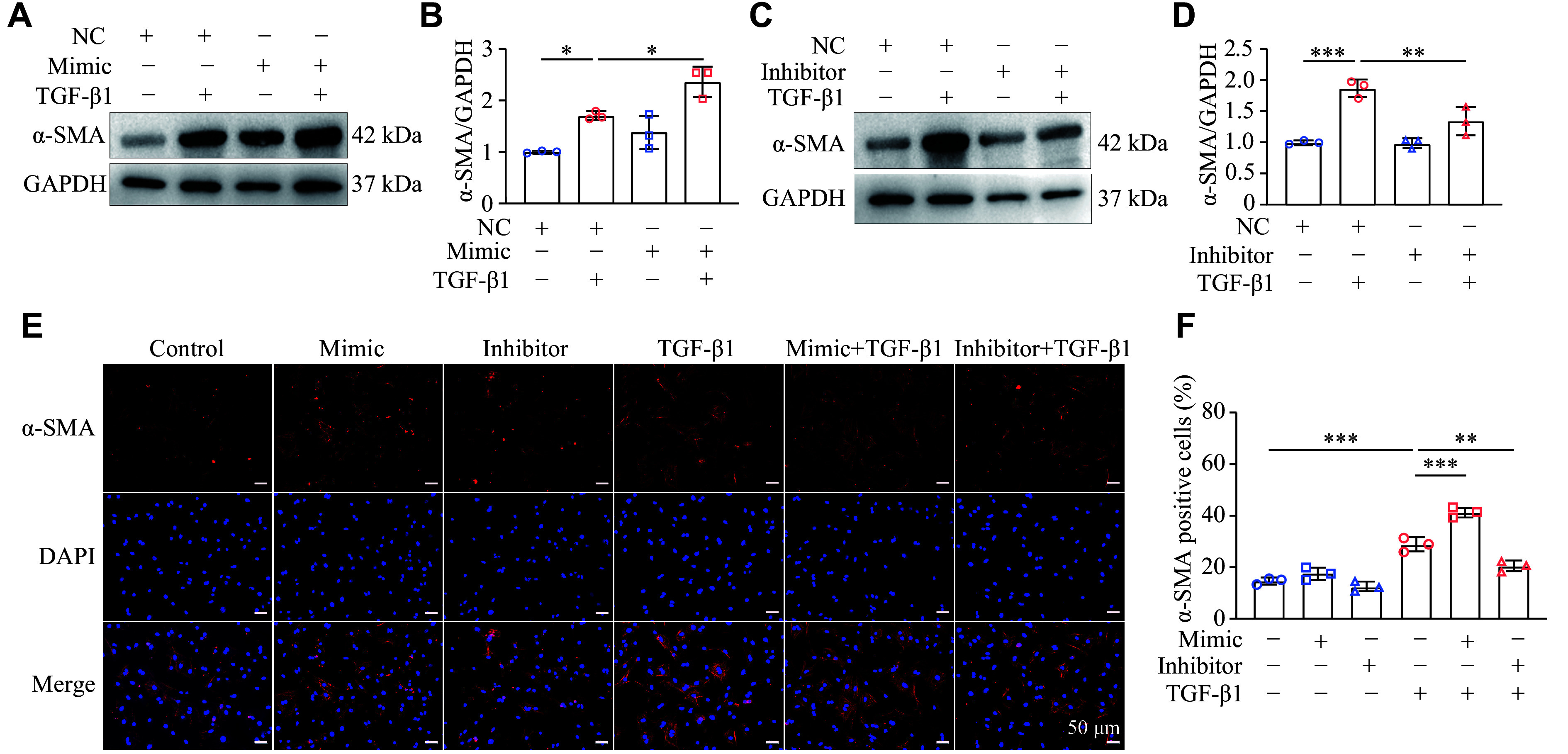
miR-19a-3p was involved in the TGF-β1-induced NRCF differentiation. The miR-19a-3p mimic or inhibitor was transfected into NRCFs for 48 h. The transfected cells were cultured in DMEM containing 1% FBS before being incubated for a further 24 h with or without 10 ng/mL TGF-β1. A–D: The levels of α-SMA following transfection with miR-19a-3p mimic (A and B) or miR-19a-3p inhibitor (C and D) were detected by Western blotting. The grayscale values of protein bands were statistically analyzed and normalized to the internal control, GAPDH. *n* = 3. E: Immunofluorescent expression of α-SMA in NRCFs. Scale bar, 50 μm. F: The extent of NRCF activation, as indicated by the proportion of cells with positive α-SMA. *n* = 3. All data are presented as the mean ± standard deviation. Statistical analyses were performed by one-way ANOVA followed by Tukey's tests for multiple comparisons. ^*^P < 0.05, ^**^P < 0.01, and ^***^P < 0.001. Abbreviations: TGF-β1, transforming growth factor β1; NRCF, newborn rat ventricular cardiac fibroblast; α-SMA, α-smooth muscle actin; GAPDH, glyceraldehyde 3-phosphate dehydrogenase; DAPI, diamidino-phenyl-indole.

Because synthetically active fibroblasts express α-SMA^[18]^, we hypothesized that overexpression of miR-19a-3p might facilitate TGF-β1-induced NRCF activation. Following the TGF-β1 administration, the protein levels of α-SMA in NRCFs were assessed by Western blotting. The results showed that miR-19a-3p mimic transfection significantly increased the protein levels of α-SMA in response to TGF-β1 stimulation (***Fig. 2A*** and ***2B***), while miR-19a-3p inhibitor transfection attenuated the effects of TGF-β1 (***Fig. 2C*** and ***2D***). Consistently, there was an increase in the number of α-SMA positive NRCFs with transfection of miR-19a-3p mimic, as evidenced by immunofluorescence; however, miR-19a-3p inhibition reduced this effect (***Fig. 2E*** and ***2F***). Additionally, the immunofluorescence results obtained from the first-passage cells were consistent with those from the second-passage cells (***Supplementary Fig. 1***, available online). Therefore, these results indicate that miR-19a-3p plays a crucial role in promoting TGF-β1-induced NRCF differentiation.

### Inhibition of miR-19a-3p restrained the TGF-β1-induced NRCF proliferation, migration, and collagen Ⅰ/Ⅲ expression

**Figure 3:**
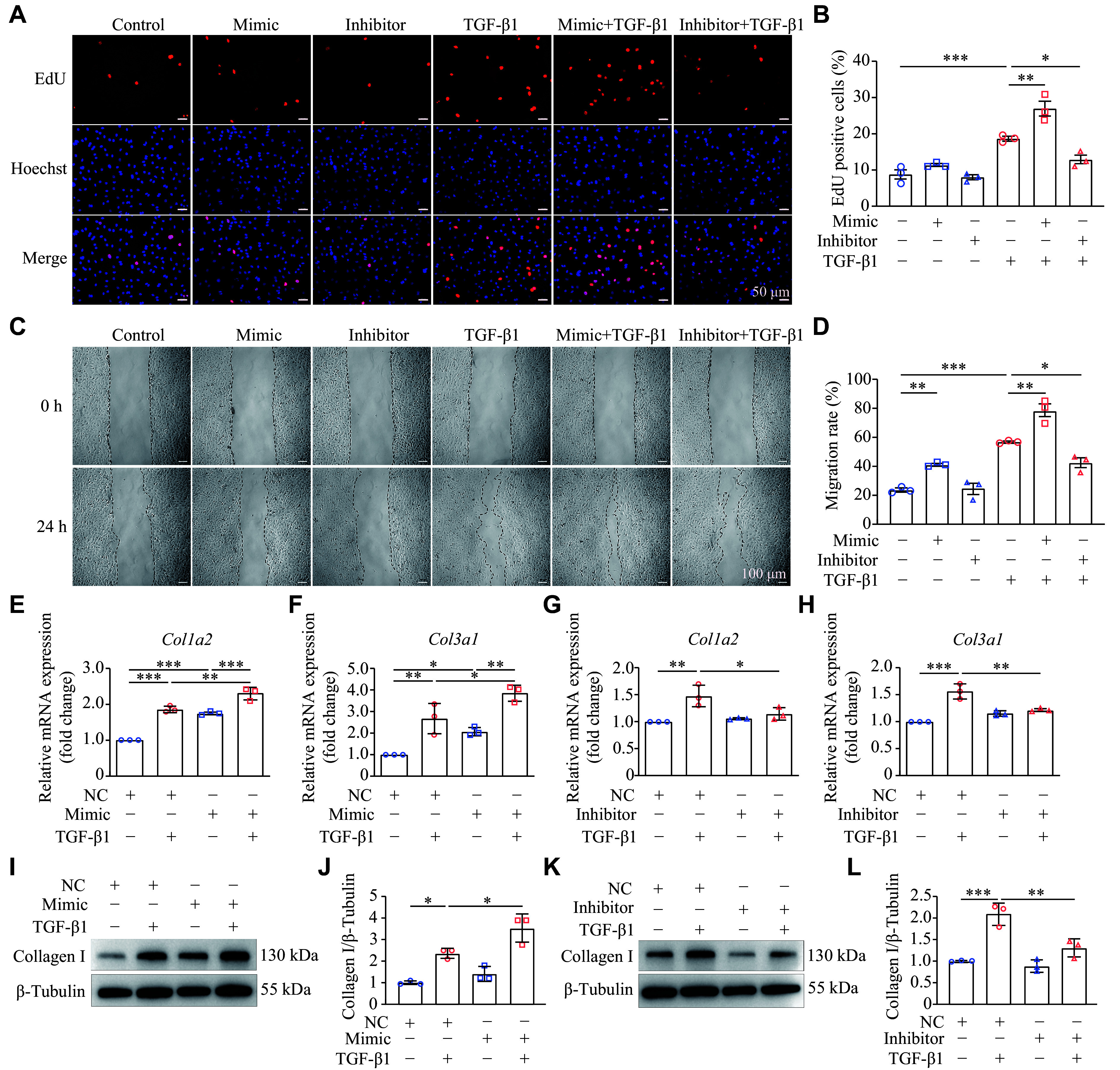
Down-regulation of miR-19a-3p restricted TGF-β1-induced NRCF proliferation, migration, and collagen deposition. NRCFs were transfected with miR-19a-3p mimic or inhibitor for 48 h, and then cells were stimulated with or without 10 ng/mL TGF-β1 for an additional 24 h. A: The proliferation of NRCFs was investigated by the EdU incorporation assay. Scale bar, 50 μm. B: The histogram displayed the proliferation rates as indicated by the proportion of EdU-positive cells. *n* = 3. C: Measurement of NRCF migration by the wound closure assay. Scale bar, 100 μm. D: The degree of NRCF motility was displayed by a histogram of the migration rate. *n* = 3. E–H: Quantitative reverse transcription-PCR was performed to examine the expression levels of fibrotic markers including *Col1a2* (E and G) and *Col3a1* (F and H). *Hprt* served as the internal reference. *n* = 3. I–L: Western blotting was used to assess the level of collagen Ⅰ following the transfection with miR-19a-3p mimic (I and J) or miR-19a-3p inhibitor (K and L). Grayscale values of the protein bands were statistically analyzed. *n* = 3. Data are presented as mean ± standard deviation. Statistical analyses were performed by one-way ANOVA followed by Tukey's tests for multiple comparisons. ^*^P < 0.05, ^**^P < 0.01, and ^***^P < 0.001*.* Abbreviations: TGF-β1, transforming growth factor β1; NRCF, newborn rat ventricular cardiac fibroblast; EdU, 5-Ethynyl-2-deoxyuridine.

We used the EdU incorporation assay to assess the effect of miR-19a-3p on NRCF proliferation. The results showed that TGF-β1 (10 ng/mL) significantly promoted NRCF proliferation. Compared with the TGF-β1 treatment alone, overexpression of miR-19a-3p further significantly accelerated NRCF proliferation, while miR-19a-3p inhibition exhibited an opposite effect (***Fig. 3A*** and ***3B***). We also examined the role of miR-19a-3p in NRCF migration using wound healing tests. TGF-β1 treatment enhanced cell migration, and overexpression of miR-19a-3p further significantly increased cell motility, while miR-19a-3p inhibition limited cell migration toward the wound (***Fig. 3C*** and ***3D***). These findings indicate that reduced miR-19a-3p levels inhibit the proliferation and migration of NRCFs *in vitro*.

Based on our findings, we hypothesized that miR-19a-3p might be involved in TGF-β1-induced collagen expression. With miR-19a-3p mimic transfection, the expression levels of collagen synthesis genes (*Col1a2* and *Col3a1*) were significantly increased in TGF-β1-stimulated NRCFs (***Fig. 3E*** and ***3F***). In contrast, miR-19a-3p inhibition reduced the mRNA levels of *Col1a2* and *Col3a1* in TGF-β1-stimulated NRCFs (***Fig. 3G*** and ***3H***). Consistently, miR-19a-3p mimic transfection further increased the protein levels of collagen Ⅰ in TGF-β1-stimulated NRCFs, while miR-19a-3p inhibitor showed an opposite effect (***Fig. 3I***–***3L***). Together, these findings imply that miR-19a-3p may play a role in TGF-β1-induced expression of collagen genes.

### miR-19a-3p enhanced the TGF-β1/SMAD2/3 signaling by targeting BAMBI

**Figure 4:**
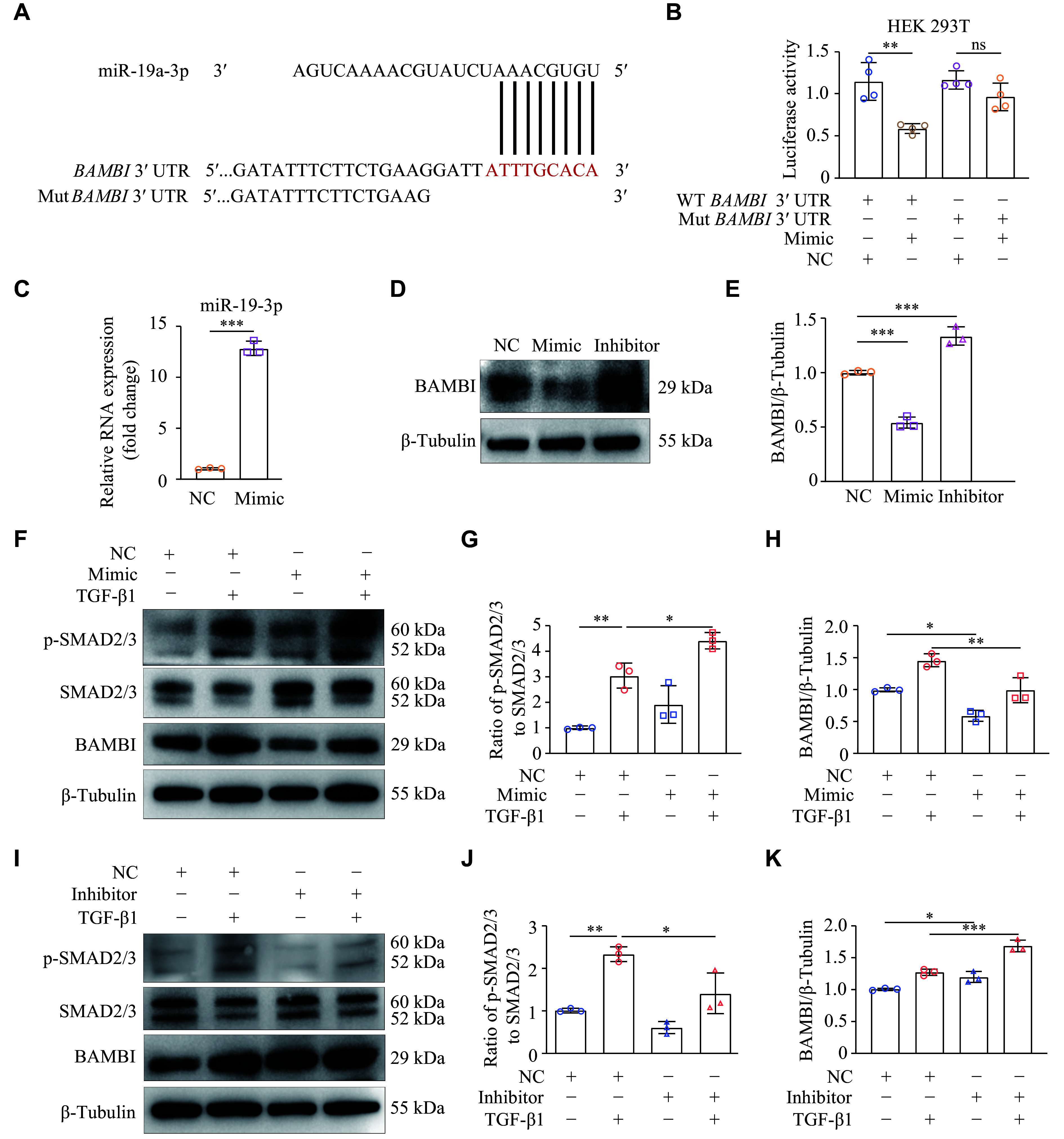
miR-19a-3p enhanced the TGF-β1 signaling by targeting BAMBI. A: TargetScanHuman 8.0 predicted miR-19a-3p binding sites in the *BAMBI* 3ʹ UTR. B: The pGL3-luciferase reporter vectors carrying either wild-type (WT) or mutant (Mut) *BAMBI* 3ʹ UTR were transfected into HEK 293T cells. Further treatment for the cells included either miR-19a-3p mimic or a negative control (NC). The activity of luciferase was detected 24 h later. *n* = 4. C–E: NRCFs were transfected with miR-19a-3p mimic or inhibitor. The levels of miR-19a-3p were detected by quantitative reverse transcription-PCR, using *U6* as the internal reference (C). *n* = 3. Western blotting was performed to examine the BAMBI protein levels (D). E: Quantitative analysis of the protein expression levels in panel D using GAPDH as an internal reference. *n* = 3. F–K: NRCFs were transfected with either miR-19a-3p mimic or its inhibitor. After 48 h of transfection, the cells were treated with TGF-β1 for an additional 24 h. The protein levels of p-SMAD2/3, SMAD2/3, and BAMBI were detected by Western blotting and were quantitatively analyzed with β-tubulin as the internal reference. *n* = 3. Data are presented as mean ± standard deviation. Statistical analyses were performed by Student's *t*-test for comparison between the two groups and one-way ANOVA followed by Tukey's tests for multiple comparisons. ^*^P < 0.05, ^**^P < 0.01, and ^***^P < 0.001. Abbreviations: TGF-β1, transforming growth factor β1; BAMBI, bone morphogenetic protein and activin membrane-bound inhibitor; ns, no significance.

Because miR-19a-3p was involved in TGF-β1-induced cardiac fibroblast activation, we performed bioinformatic analysis using TargetScanHuman 8.0 to determine the precise target sites of miR-19a-3p. A highly conserved miR-19a-3p binding site was discovered in the 3′ UTR of *BAMBI* mRNA across species (***Fig. 4A***; ***Supplementary Fig. 2***, available online). We then performed a dual luciferase reporter assay to determine if the anticipated binding sites in the 3′ UTR of *BAMBI* were necessary for the regulatory function of miR-19a-3p. Strikingly, luciferase activity was significantly decreased when the miR-19a-3p mimic was overexpressed in HEK293T cells that had been transfected with the WT *BAMBI* 3′ UTR, which contains miR-19a-3p binding sites (***Fig. 4B***). In contrast, luciferase activity was not affected in HEK293T cells transfected with a mutant *BAMBI* 3′ UTR, in which the predicted miR-19a-3p binding sites were altered (***Fig. 4B***).

To further investigate the role of miR-19a-3p in regulating BAMBI, we examined BAMBI protein levels in NRCFs after transfection with the miR-19a-3p mimic or inhibitor. We found that BAMBI levels were significantly reduced when the miR-19a-3p mimic was overexpressed, but increased following inhibition of miR-19a-3p (***Fig. 4C***–***4E***). These results demonstrate that miR-19a-3p directly binds to the 3′ UTR of *BAMBI* mRNA, thereby inhibiting its expression.

Both malignant fibrosis and normal tissue healing increase TGF-β1 expression^[19]^. Once activated, TGF-β1 binds to TβR-Ⅰ and TβR-Ⅱ. SMAD2/3 are phosphorylated by TβR-Ⅰ, leading to their binding with SMAD4 and subsequent translocation into the nucleus to trigger gene transcription^[20]^. To investigate whether miR-19a-3p regulates the expression of BAMBI and TGF-β1 signaling in cardiac fibroblasts, we transfected NRCFs with miR-19a-3p mimic or NC. After 48 h of the transfection, TGF-β1 was administered, and the phosphorylation levels of SMAD2/3 were detected by Western blotting. The results showed that TGF-β1 stimulation activated SMAD2/3 phosphorylation, compared with NC. Overexpression of miR-19a-3p further increased the phosphorylation of SMAD2/3, but reduced the expression of BAMBI induced by TGF-β1 (***Fig. 4F***–***4H***). These results suggest that miR-19a-3p promotes the activation of TGF-β1/SMAD2/3 signaling pathway in cardiac fibroblasts.

To further elucidate the molecular mechanisms underlying the correlation between miR-19a-3p and TGF-β1 signaling pathway, we transfected NRCFs with miR-19a-3p inhibitor or NC. The results showed that the inhibition of miR-19a-3p significantly reduced SMAD2/3 phosphorylation following TGF-β1 stimulation (***Fig. 4I*** and ***4J***). In addition, the protein levels of BAMBI were upregulated by miR-19a-3p inhibitor (***Fig. 4K***). Collectively, these results demonstrate that miR-19a-3p activates the TGF-β1/SMAD2/3 signaling pathway by suppressing BAMBI expression.

### Overexpression of BAMBI ameliorated the miR-19a-3p-induced fibroblast collagen expression through TGF-β1 signaling

**Figure 5:**
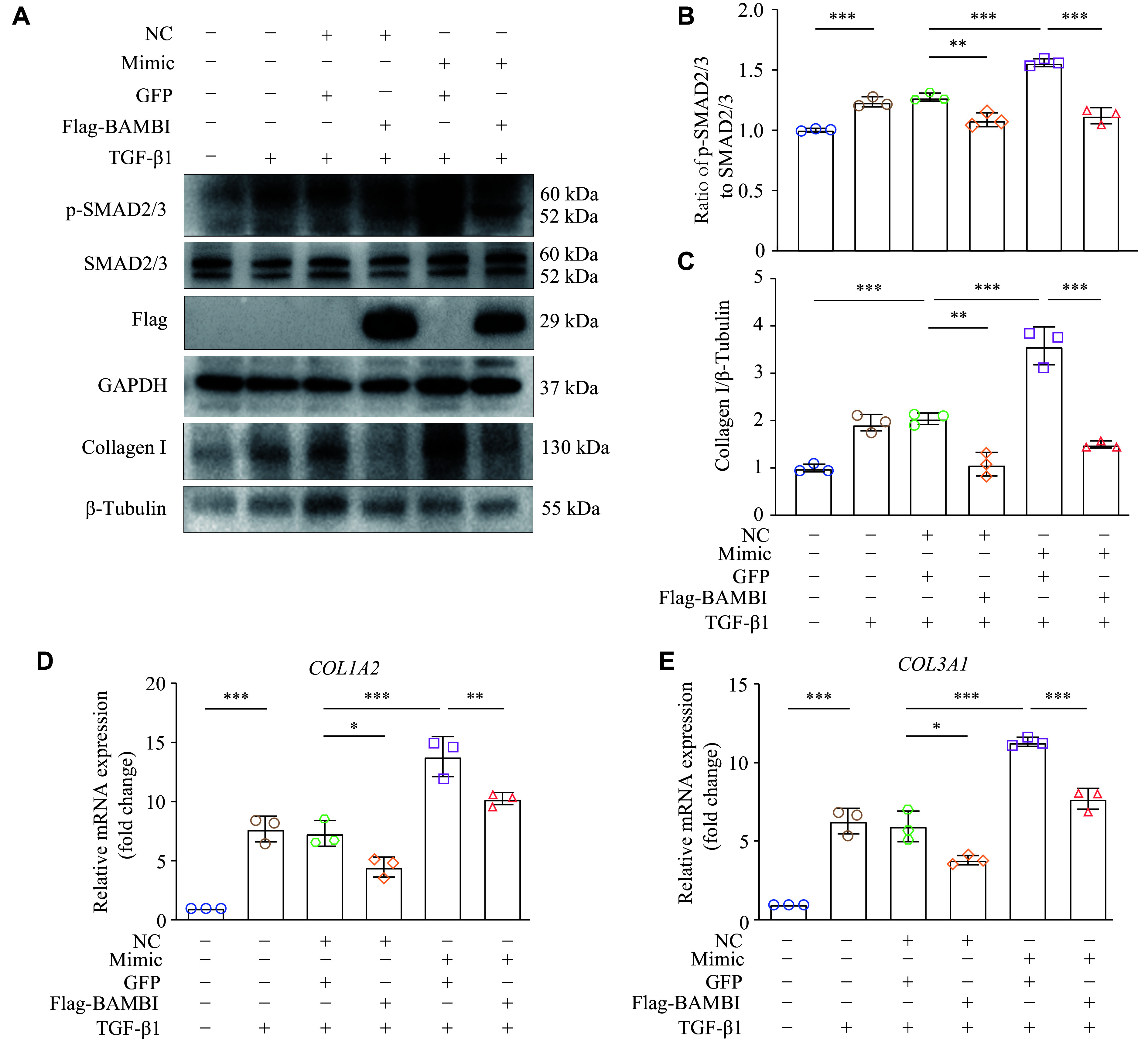
Elevated BAMBI ameliorated miR-19a-3p-induced fibroblast collagen expression through TGF-β1 signaling. In addition to miR-19a-3p mimic or negative control (NC), pcDNA3.1-BAMBI-3×Flag or pcDNA3.1-GFP were transfected into HT1080 cells. After 48 h of transfection, the cells were treated with TGF-β1 for an additional 24 h. A: Western blotting was used to identify the protein levels of p-SMAD2/3, SMAD2/3, and Collagen Ⅰ. B: SMAD2/3 was used as the internal reference to quantify the level of p-SMAD2/3. C: Grayscale values for collagen Ⅰprotein were statistically analyzed using β-tubulin as the internal reference. D and E: Quantitative reverse transcription-PCR was performed to detect the expression levels of *COL1A2* and *COL3A1*. *HPRT* was used as the internal reference gene. *n* = 3. Data are presented as mean ± standard deviation. Statistical analyses were performed by one-way ANOVA followed by Tukey's tests for multiple comparisons. ^*^P < 0.05, ^**^P < 0.01, and ^***^P < 0.001. Abbreviations: TGF-β1, transforming growth factor β1; BAMBI, bone morphogenetic protein and activin membrane-bound inhibitor.

To demonstrate the role of BAMBI in TGF-β1/SMADs cascade, we transfected miR-19a-3p mimic into HT1080 cells overexpressing BAMBI, and then detected the phosphorylation of SMAD2/3. The results showed that overexpression of BAMBI significantly reduced the miR-19a-3p mimic-induced phosphorylation of SMAD2/3 (***Fig. 5A*** and ***5B***), providing further evidence that miR-19a-3p stimulates TGF-β1/SMAD2/3 signaling by inhibiting BAMBI.

The main characteristic of fibroblast activation is the increased ECM expression, such as collagen. To further validate whether BAMBI participates in miR-19a-3p-mediated collagen gene expression, we co-transfected HT1080 cells with miR-19a-3p mimic or its NC, along with pcDNA3.1-3×Flag-BAMBI or pcDNA3.1-GFP, with or without TGF-β1 treatment. The results showed that overexpression of BAMBI significantly reduced the protein levels of collagen Ⅰ in HT1080 cells stimulated with TGF-β1 when co-transfected with miR-19a-3p mimic (***Fig. 5A*** and ***5C***), as did the mRNA levels of *COL1A2* and *COL3A1* (***Fig. 5D*** and ***5E***). These results demonstrate that BAMBI attenuates miR-19a-3p-mediated collagen gene expression.

## Discussion

**Figure 6:**
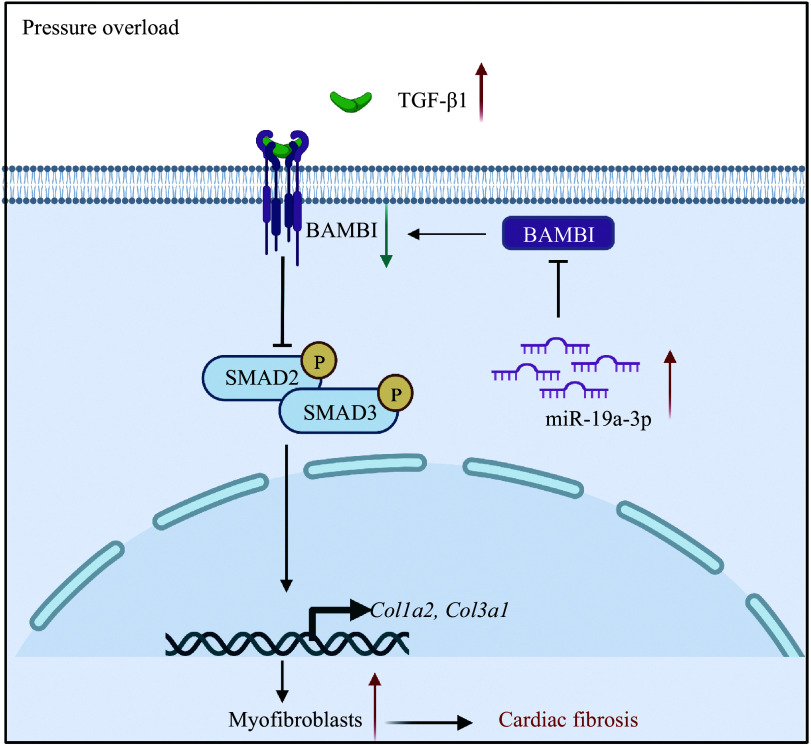
miR-19a-3p enhances TGF-β1-induced cardiac fibroblast activation *via* targeting BAMBI. BAMBI acts as a pseudo receptor of TGF-β1 to block the phosphorylation of SMAD2/3. TGF-β1 increases miR-19a-3p expression, which suppresses BAMBI and promotes the phosphorylation of SMAD2/3 and cardiac fibroblast activation under pressure overload. Abbreviations: TGF-β1, transforming growth factor β1; BAMBI, bone morphogenetic protein and activin membrane-bound inhibitor.

In the current study, we observed that miR-19a-3p expression was increased in the hearts of both patients with cardiac hypertrophy and mice subjected to TAC. We further verified that TGF-β1 upregulated the expression of miR-19a-3p, which inhibited the expression of BAMBI *via* post-transcriptional regulation in NRCFs, ultimately promoting the activation of TGF-β1/SMAD2/3 and enhancing cardiac fibroblast activation (***Fig. 6***).

Left ventricular pathological fibrosis is likely induced by pressure overload^[21]^. The most significant aspect in the pathological phase of myocardial fibrosis is the activation of cardiac fibroblasts. Increased interstitial collagen deposition, myocardial systolic and diastolic dysfunction are caused by the stress-induced conversion of cardiac fibroblasts into myofibroblasts^[22]^. TGF-β1 is an important factor that promotes ECM deposition, and its expression is elevated when the heart experiences pressure overload^[23]^. Consistently, we demonstrated the elevated TGF-β1 expression following the TAC surgery in mice. The TGF-β1/SMAD3 signaling pathway is important for fibroblast differentiation^[24]^. α-SMA, a key source of ECM proteins in cardiac remodeling, is expressed by myofibroblasts, which differ from fibroblasts physically and functionally and represent a manifestation of the cardiac fibrotic response. Similarly, the TGF-β1 signaling promotes the ECM formation, including collagen, further accelerating the onset of ventricular fibrosis^[25]^. Our *in vitro* experiments using NRCFs demonstrated the activation of p-SMAD2/3 and an increase in collagen expression upon TGF-β1 stimulation.

MicroRNAs have been implicated in fibrotic processes in recent years. For instance, the inhibition of miR-221-3p/miR-222-3p has been shown to accelerate TGF-β-induced myocardial fibrosis^[26]^, while several studies have indicated that microRNAs modulate the TGF-β signaling pathway in fibrosis^[27]^.

In the current study, we found that TGF-β1 upregulated the expression levels of miR-19a-3p both *in vivo* and *in vitro*. However, Zou *et al*^[28]^ reported that miR-19a-3p/19b-3p were expressed at low levels in the plasma of heart failure patients and that miR-19a-3p inhibited autophagy *via* the TGF-β signaling pathway in a human cardiac fibroblast cell line. The plasma level of miR-19a-3p may be influenced by multiple organs, including the liver and kidney; however, its expression in cardiac tissues has rarely been reported. We demonstrated the increased levels of miR-19a-3p in the cardiac tissues of both patients with cardiac hypertrophy and mice following the TAC surgery, as well as in TGF-β1-induced neonatal rat cardiac fibroblasts *in vitro*. Additionally, Zhang *et al*^[29]^ demonstrated that TGF-β increased the expression of miR-19, which promoted the development of renal fibrosis by inhibiting PTEN. The TGF-β1/SMADs signaling pathway has been implicated in cardiac fibrosis^[5]^. Consistently, we observed that the transfection of miR-19a-3p mimics significantly activated cardiac fibroblasts. Collectively, the current study provides the first evidence that miR-19a-3p facilitates cardiac fibroblast differentiation, proliferation, and collagen gene expression by the TGF-β1/SMAD2/3 signaling pathway.

Through directly binding to the 3′ UTR of their target mRNAs, microRNAs normally inhibit gene expression at the post-transcriptional level^[30]^. In the current study, we identified the miR-19a-3p binding site in the 3′ UTR of *BAMBI* mRNA and demonstrated the post-transcriptional regulation of miR-19a-3p on BAMBI using a luciferase reporter gene assay. BAMBI is a transmembrane protein that inhibits TGF-β1 signaling by lacking an intracellular kinase domain but having an extracellular domain similar to the TβR-Ⅰ^[31]^. Endogenous BAMBI may bind to TβR-Ⅱ, resulting in a decrease in both the TβR-Ⅰ/TβR-Ⅱcomplex and the quantity of phosphorylated TβR-Ⅰ in this complex^[32]^. These processes may account for the ability of BAMBI to block the TGF-β signaling, although their interaction with type Ⅱ receptors has not been ruled out^[32]^. BAMBI has also been demonstrated to suppress β-catenin and TGF-β1 signaling pathways in hepatocellular carcinoma cells^[33]^. Villar *et al*^[34]^ observed that TGF-β1 stimulation increased BAMBI expression in primary mouse fibroblasts *via* a positive feedback; furthermore, BAMBI deletion exacerbated the TGF-β1-induced profibrotic response in primary cardiac fibroblasts, whereas BAMBI overexpression alleviated this response in NIH-3T3 fibroblasts. Interestingly, we found that the transfection of miR-19a-3p mimic in NRCFs inhibited BAMBI expression and upregulated the levels of p-SMAD2/3, resulting in further increases in *Col1a2* and *Col3a1* mRNA levels. Consistently, this effect was reversed upon the inhibition of miR-19a-3p. Therefore, our findings indicate that miR-19a-3p participates in the regulation of the TGF-β1 signaling pathway by inhibiting the expression of BAMBI.

In summary, the expression level of miR-19a-3p was upregulated in the myocardial tissues of cardiac fibrosis. TGF-β1 induces the high expression of miR-19a-3p in NRCFs. Moreover, miR-19a-3p enhances TGF-β1-induced differentiation and proliferation of cardiac fibroblasts by targeting BAMBI. Thus, miR-19a-3p may serve as a promising biomarker and therapeutic target for myocardial fibrosis.

## SUPPLEMENTARY DATA

Supplementary data to this article can be found online.
