# Supplementary material for: miR-19a-3p enhances TGF-β1-induced cardiac fibroblast activation via targeting BAMBI
Source: J Biomed Res. 2024 May 29;39(2):171–83. doi: 10.7555/JBR.37.20230313 (PMC11982684; doi:10.7555/JBR.37.20230313)
Supplement: Supplementary file 1 — Supplementary data to this article can be found online. [file jbr-39-2-171-S1.pdf]

Available online at www.jbr-pub.org.cn

Open Access at PubMed Central

Journal of Biomedical Research, 2025 39(2): 1–2

Supplementary Data

JBR

miR-19a-3p augments TGF-β1-induced cardiac fibroblast activation *via* targeting BAMBI

Pengxi Shi[1], Ao Tan[1], Yuanyuan Ma[1], Lingli Que[1], Chuanfu Li[2], Yongfeng Shao[3], Haoliang Sun[3,✉], Yuehua Li[1,✉], Jiantao Li[1,✉]

[1]Key Laboratory of Targeted Intervention of Cardiovascular Disease, Collaborative Innovation Center for Cardiovascular Disease Translational Medicine, School of Basic Medical Science, Nanjing Medical University, Nanjing, Jiangsu 211166, China;
[2]Department of Surgery, East Tennessee State University, Johnson City, TN 37614-0575, USA;
[3]Department of Cardiovascular Surgery, the First Affiliated Hospital of Nanjing Medical University, Nanjing, Jiangsu 210029, China.

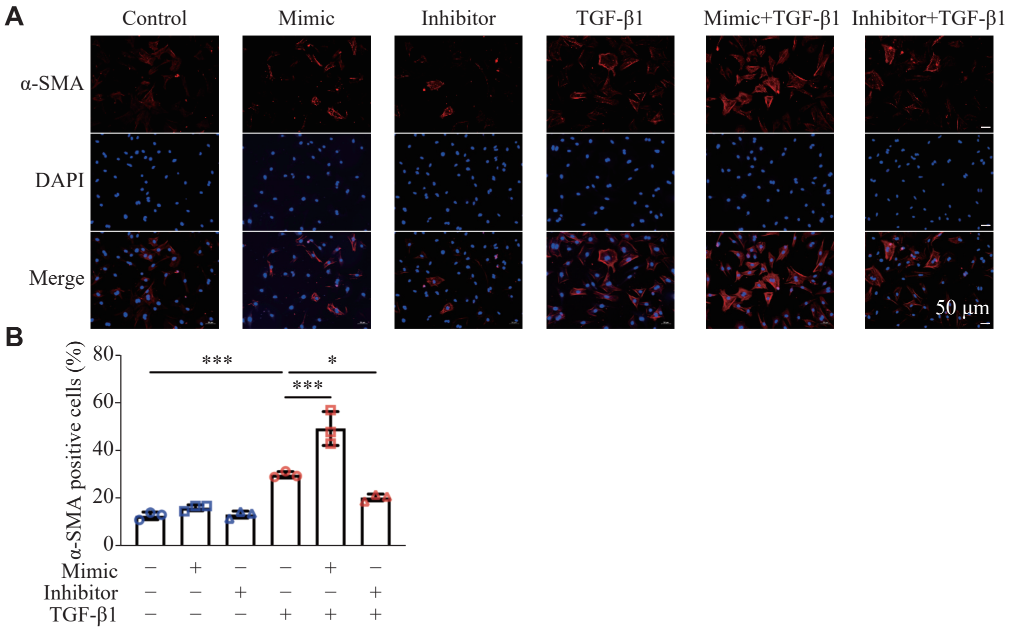

Supplementary Fig. 1 **Immunofluorescence of the first passage of newborn rat ventricular cardiac fibroblasts (NRCFs).** The miR-19a-3p mimic or inhibitor was transfected into the first passage of NRCFs for 48 h. Transfected cells were cultured in DMEM containing 1% FBS before being incubated for a further 24 h with or without TGF-β1 (10 ng/mL). A: Immunofluorescence staining of α-SMA. Scale bar, 50 μm. B: The extent of NRCF activation indicated by the proportion of α-SMA positive cells. $n = 3$. Data are presented as mean ± standard deviation. Statistical analyses were performed by one-way ANOVA followed by Tukey's tests for multiple comparisons. $^{*}P < 0.05$ and $^{***}P < 0.001$. Abbreviations: TGF-β1, transforming growth factor β1; α-SMA, α-smooth muscle actin; DAPI, diamidino-phenyl-indole.

✉Corresponding authors: Haoliang Sun, Department of Cardiovascular Surgery, the First Affiliated Hospital of Nanjing Medical University, 300 Guangzhou Road, Nanjing, Jiangsu 210029, China. E-mail: sunhaoliang@jsph.org.cn; Jiantao Li and Yuehua Li, Key Laboratory of Targeted Intervention of Cardiovascular Disease, Collaborative Innovation Center for Cardiovascular Disease Translational Medicine, School of Basic Medical Science, Nanjing Medical University, 101 Longmian Avenue, Nanjing, Jiangsu 211166, China. E-mails: ljt@njmu.edu.cn (Jiantao Li) and yhli@njmu.edu.cn (Yuehua Li).

Received: 18 December 2023; Revised: 23 April 2024; Accepted: 30 April 2024; Published online: 29 May 2024

CLC number: R542.23, Document code: A

The authors reported no conflict of interests.

This is an open access article under the Creative Commons Attribution (CC BY 4.0) license, which permits others to distribute, remix, adapt and build upon this work, for commercial use, provided the original work is properly cited.

© 2025 by Journal of Biomedical Research.

https://doi.org/10.7555/JBR.37.20230313

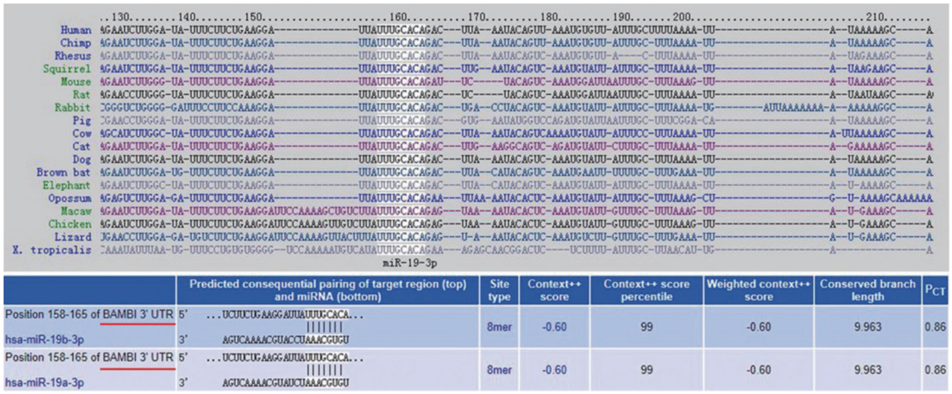

Supplementary Fig. 2 **miR-19a-3p binding sites in the *BAMBI* 3′ UTR.** TargetScanHuman 8.0 predicted miR-19a-3p binding sites in the *BAMBI* 3′ UTR, and they were highly conserved among species.
